# Perinatal exposure to the environmental pollutant butyl benzyl phthalate (BBP) induces sex-specific alterations in the neuro-immune-endocrine network in adult rats

**DOI:** 10.3389/fimmu.2026.1838242

**Published:** 2026-08-12

**Authors:** Diana Lizeth Ruiz-Antonio, Karen E. Nava-Castro, Claudia Angelica Garay-Canales, Yair Rodríguez Santiago, María Del Sol Ríos-Avila, César Antonio Zavala-Lopez, Lenin Pavón, Gilberto Pérez-Sánchez, Lilia Diaz, Jorge Morales-Montor

**Affiliations:** 1 Laboratorio de Neuroinmunoendocrinologia, Instituto de Investigaciones Biomedicas, Departmento de Inmunologia, Universidad Nacional Autonoma de Mexico, Mexico, Mexico; 2 Posgrado en Ciencias Bioquimicas, Unidad de Posgrado, Universidad Nacional Autonoma de Mexico, Mexico, Mexico; 3 Laboratorio de Biologıa y Quımica Atmosferica, Departamento de Ciencias Ambientales, Instituto de Ciencias de la Atmosfera y Cambio Climatico, Universidad Nacional Autonoma de Mexico, Mexico, Mexico; 4 Posgrado en Ciencias Biologicas, Unidad de Posgrado, Universidad Nacional Autonoma de Mexico, Mexico, Mexico; 5 Laboratorio de Psicoinmunología, Departamento de Psicoinmunología, Instituto Nacional de Psiquiatría “Ramon de la Fuente Muñiz”, Mexico, Mexico

**Keywords:** butyl benzyl phthalate BBP, cytokines, development, endocrine disruptor, neuro-immune-endocrine NIE network, neurotransmitters, steroid hormones, perinatal exposure

## Abstract

The plasticizer butyl benzyl phthalate (BBP), a ubiquitous endocrine disruptor, is commonly used in PVC products and can leach into the environment, leading to human exposure. BBP has been detected in the placenta, amniotic fluid, and breast milk, indicating potential exposure during gestation and lactation. BBP is an endocrine disruptor compound (EDC) with estrogenic and anti-androgenic activity, which, at high or acute doses, may interfere with the development of hormone-dependent systems, including components of the neuro-immune-endocrine network (NIE) and sexual dimorphism. This study aimed to investigate the effects of perinatal BBP exposure on various components of the NIE network in adult male and female rats. Pregnant rats were administered drinking water with or without BBP (50 μg/L) from gestational day 5 until weaning. Offspring were assessed at 9 weeks of age by examining sex hormone levels in serum, immune cell populations in the spleen, neurotransmitters in the hippocampus, and cytokines in serum, hippocampus, and spleen. Results showed that BBP exposure led to lower weaning weights in both sexes, with persistent effects in males. Splenic T and T-helper lymphocyte populations increased in both sexes. In the hippocampus, females exhibited elevated proinflammatory cytokines, while males showed decreased serotonin levels, indicating sex-specific alterations in hormone, cytokine, and neurotransmitter levels in adult offspring.

## 1 Introduction

Plasticizers are a threat to humans, animals, and the environment (1–3). Over the past few decades, the production of plastic has grown significantly, with almost 80% of it ending up in landfill sites (4). Plasticizers are additives used to adapt plastic materials for many different uses. Phthalates are one of the most common types of plasticizers. They make plastic more malleable, flexible, and durable. Phthalates are used in PVC, paints, resins, hygiene, and beauty products (5, 6). However, phthalates do not form covalent bonds with the polymer matrix, meaning they can easily leach after changes in pH or temperature (7).

We are constantly exposed to phthalates by different routes, including oral, dermal, and inhalation (8–10). Butyl benzyl phthalate (BBP) is a high-molecular-weight phthalic acid ester with low but considerable water solubility (3.8 mg/L) (7), and it has been found in surface and ground waters in concentrations ranging from 1 to 201 ng/L (11) as well as in bottled water at concentrations of up to 12.1 µg/L (12). It is also found in many foods, such as milk, bottled water, alcoholic beverage, edible oils, pork and chicken (13), and it has been estimated that we consume up to 15.5 μg/kg BBP daily in our diet (14, 15).

Exposure to phthalates begins even before birth. The presence of phthalates in amniotic fluid, the placenta, and meconium (16–19) suggests that maternal exposure allows fetal exposure due to these chemicals are capable to cross placental barrier. During breastfeeding, exposure continues through breast milk and infant formula (20, 21) in addition to the use of baby bottles and hygiene products (22, 23). Thus, exposure is facilitated during critical periods of development, temporary windows of high vulnerability in which external agents can alter cellular programming processes, generating long-term alterations.

Phthalates, such as BBP, are endocrine disruptor compounds (EDCs). BBP possesses estrogenic agonism as it has affinity to the estrogen receptors (ER), ERα and ERβ (24). Through this activation, BBP promotes cancer development in breast cancer models (25). In addition, BBP has been shown to have anti-androgenic effects in experimental studies on male rodents. These rodents were exposed to BBP during gestation, and the results showed reproductive system malformations, including shortened anogenital distance (AGD), hypospadias, cryptorchidism, and gubernacular lesions (26–29). Moreover, BBP decreases testosterone production (30, 31), increases FSH release (28) and affects sperm quality (32), potentially leading to fertility issues.

As an endocrine disruptor, BBP has the ability to interfere with the development of hormone-dependent systems, such as the neuro-immune-endocrine network (NIE) and sexual dimorphism (33). In mice, ERs are expressed and active during embryo development since the 13.5 day (34). The ER activity is higher in ectoderm-derived tissues, including the brain and skin. The gonad tissue ER activity is low until the last days of gestation, following gestational day 18.5. Simultaneously, testosterone plays a key role during the so-called masculinization programming window, around 15.5 and 18.5 gestation days (35, 36). Additionally, sexual hormones and estrogen receptors fluctuate significantly during pregnancy and labor (37, 38). Thus, there are multiple tissues and processes in which BBP can interfere during the perinatal stage.

Although the masculine reproductive system has been the most studied system affected by BBP and other phthalates, there is evidence of adverse effects on female reproductive health, including disruption of ovarian function, structural damage to reproductive organs, and altered hormone levels (39–41). Regarding the immune and nervous systems, some correlations have been shown. BBP exposure increases the risk of developing asthma and eczema by promoting the Th2 type inflammation (42–44), alters the brain metabolomics in a sexually dimorphic way (45), induces behavioral changes, modifies the expression of hormone receptors in some cerebral areas (46), decreases hippocampal serotonin, causes oxidative stress, and worsens performance on spatial memory tasks (47, 48).

Currently, the European Chemicals Agency (ECHA) recognizes the No observed adverse effect level (NOAEL) of BBP at 50 mg/kg bw/day for developmental effects and the oral NOAEL of 0.5 mg/kg/day. In contrast, globally, daily exposure to BBP is reported to range from 0.009 to 15.53 μg/kg bw/day (14, 49). Although reported exposure levels are much lower than those considered safe, several correlations have been found between phthalate exposure and health effects, including alterations in the NIE network (1). Nevertheless, the novelty of our study lies in addressing experimental data on BBP biological effects are remarkably limited. Moreover, to the best of our knowledge, no previous study has examined BBP from the integrative perspective of the neuroimmunoendocrine network—a framework that is particularly relevant given the compounds known endocrine-disrupting properties and their potential crosstalk with immune and neural systems. Furthermore, the sexually dimorphic dimension of BBP exposure has received no attention in the existing literature, despite sex being a critical variable in endocrine disruption research.

Therefore, there is a growing interest in understanding the effects of chronic low-dose exposure to plasticizers, especially at the cellular and molecular level. The present study aims to investigate the dimorphic effect of perinatal chronic exposure to a low dose of BBP in rats on soluble factors that enable communication between the nervous, endocrine, and immune systems.

## 2 Materials and methods

### 2.1 Animals

The care and experimental practices with the animals were carried out in the facilities of the Biological Models Unit (UMB), Institute of Biomedical Research (IIBo), UNAM, in adherence to Mexican standards (NOM-062-ZOO-1999) and the recommendations of the National Institutes of Health (NIH) of the United States of America (Guide for the Care and Use of Laboratory Animals). The Committee for the Care and Use of Laboratory Animals (CICUAL-IIB) permit number granted ID 6298 for the performance of this protocol.

**Figure 1:**
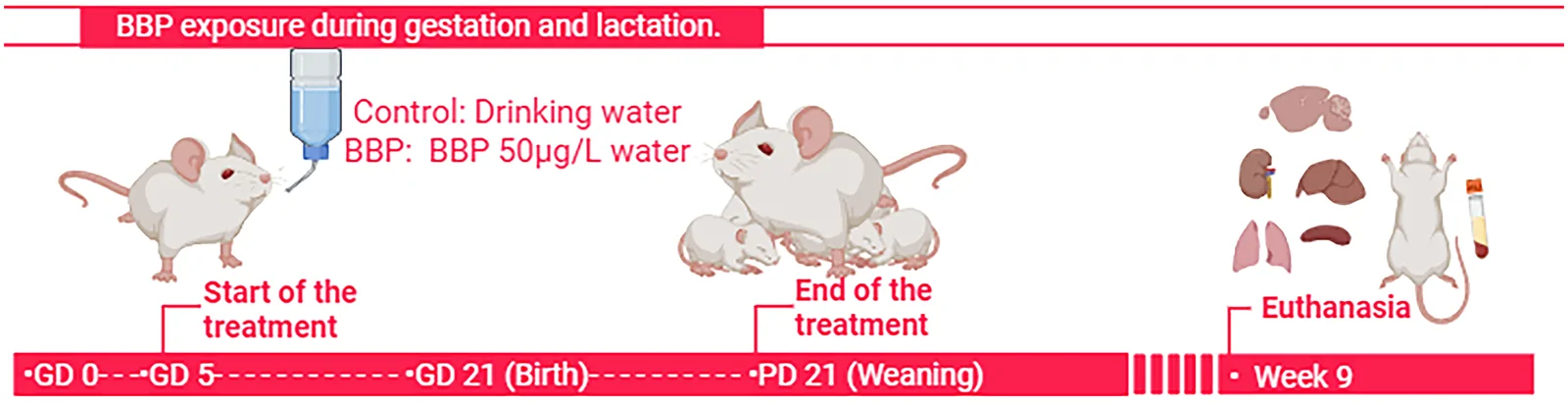
Protocol of exposure to BBP in drinking water. Pregnant Wistar rats were exposed to 50 μg/L of BBP through their drinking water during gestation and lactation. The pups were evaluated in adulthood.

We use six nine-week-old Wistar rats (three females and three males) for the gestation period. The rats were divided into two control groups (Ctrl) and one experimental group (BBP). The offspring of the control and experimental mother rats were then evaluated (**Figure 1**).

BBP (Sigma-Aldrich) was administered to the experimental group via drinking water at a concentration of 50 μg/L from gestational day 5 to postnatal day (PND) 21. The control group received drinking water without BBP under otherwise identical conditions. The water used was kept in glass bottles before and during administration to avoid plastic contamination. The daily intake was ~13 μg/kg/day and this were calculated considering that rats consumed ~73 ml of water daily.

The groups were formed as follows: 14 Ctrl males, 12 Ctrl females, 8 BBP males and 7 BBP females. At 9 weeks of age, the rats were sacrificed by guillotine. Blood, spleen, lung, liver, kidney, and brain were collected from each rat.

### 2.2 Flow cytometry assays

Splenocyte subsets were quantified by flow cytometry. The following immune cell populations were analyzed: total T cells (CD3+), helper T cells (CD3+/CD4+), cytotoxic T cells (CD3+/CD8+), B cells (CD45RA+), natural killer (NK) cells (CD161+), Tγδ+ cells, and macrophages (CD11b/c+). Biotinylated antibodies were revealed using PECy5-conjugated streptavidin. A detailed list of the antibodies used for immunophenotyping, including clone, fluorochrome, catalog number, dilution, and supplier, is provided this **Table 1**.

**Table 1:** Details for antibody used.

Target	Clone	Fluorochrome	Dilution	Catalog #	Supplier
CD3	1F4	Alexa Fluor 488	1:100	201406	Biolegend
CD4	OX-35	PECy5	1:100	554839	BD Biosciences
CD8	OX-8	PE	1:100	201706	Biolegend
CD45Ra	OX-33	PE	1:100	202307	Biolegend
CD161	10/78	Alexa Fluor 647	1:200	203110	Biolegend
Tγδ	V65	PE	1:600	202605	Biolegend
CD11b/c	OX-42	Biotin	1:400	201803	Biolegend
Streptavidin	–	PECy5	1:100	405205	Biolegend

The spleen was disaggregated using a sterile mesh of 70μm in 1.5 mL of PBS at 4 °C. The suspension was collected and centrifuged (2000 RPM, 3 min). The pellet was resuspended and washed with PBS. It was centrifuged again, and the pellet was resuspended with 500 μl of lysis buffer for 30 seconds to repeat the centrifugation and PBS wash. The final pellet was resuspended with 1mL of PBS. 50 µl of the sample was diluted in 950μl of FACS buffer. In the corresponding wells, 25 μl of each primary antibody prepared at twice the desired final concentration was added to 25 μl of the cell dilution. It was left in incubation for 30 minutes, protected from light. The plate was centrifuged at 200 RPM for 3 minutes. It was decanted and resuspended in a vortex, then we washed with 50 μl of FACS buffer and then with PBS. Finally, it was resuspended at 200 μl of PFA and stored at 4 °C. It was read in the Flow Cytometer Attune Red/Blue from the Laboratorio Nacional de Citrometria de Flujo (LabNalCit, UNAM). The analysis was performed using the software Biosciences FlowJo v10.10.

### 2.3 Hormones

Serum steroid hormones were quantified by immuno-absorbent essay with immunosorbent assay (EIA) following the instructions of manufacturer, we used the following kits: ADI-900–093 for Dehydroepiandrosterone (DHEA; Enzo, USA), EIA5761R for Dihydrotestosterone (DHT; DRG^®^, USA), EIA1561R for Progesterone (DRG^®^, USA), EIA2693 for Estradiol (DRG^®^, USA), EIA1559 for Testosterone (DRG^®^, USA) and EIA4164 for Corticosterone (DRG^®^, USA).

Before the EIA, we performed steroid extraction. The samples were centrifuged at 14600 RCF for 2 minutes. 4 mL of ether was added to the sample. After 30 seconds of agitation, they were kept at 4 °C for 60 minutes. After that time, each sample was frozen by immersion in dry ice with acetone for 20 seconds. The supernatant was recovered after the ether evaporation. The sample was resuspended with 100 μl of ethanol and 150 μl of Tris buffer. The EIA was performed following the corresponding kit instructions. Finally, they were read in the Cytation1 imaging reader, Biotek Agilent. The quantification was normalized to the total protein content per sample.

### 2.4 Cytokines

Cytokine quantification was performed according to the manufacturer’s instructions of MILLIPLEX rat cytokine kit (Rat Cytokine/Chemokine Magnetic bead panel) Cat. RECYTMAG-65K for IL-5, TNF-α, IL-1β, IL-6, IFN-γ, IL-10, and IL-4 in serum, spleen, and hippocampus samples.

Hippocampal samples were homogenized with 120 μl of buffer (Tris-HCl 20 mmol/L, NaCl 150mmol/L, PMSF 1mmol/L, Tween 20 0.05%, and 10x protease inhibitor cocktail), macerated with pistil in 1.5ml tubes, and kept on ice. They were sonicated for 1 min and vortexed for 10 sec. Then, they were incubated for 10 minutes and centrifuged for 15 minutes at 15000 RPM at 4 °C. The supernatant was collected and stored at -20 °C. Spleen was also macerated with pistils in 1.5ml tubes with 50 mM Tris-HCl buffer. 1μl was taken to calculate the amount of protein by the Bradford protocol. Before placing them on the plate, they were diluted, adding 10μl of sample and 30μl of the corresponding buffer, then centrifuged for 2 minutes at 10000 rpm at 4 °C. The plates were read in the MAGPIX equipment of the Millipore brand and analyzed in the program Belysa™ Immunoassay Curve Fitting Software (Merck, Germany), provided by the Unidad de Investigación de la Facultad de Veterinaria y Zootecnia (UNAM).

### 2.5 Neurotransmitters

In hippocampal samples, tryptophan (Trp), serotonin (5-HT), 5-Hydroxy indoleacetic acid (5-HIAA), dopamine (DA), and 3,4-Dihydroxyphenylacetic acid (DOPAC) were measured. Quantification was performed with HPLC equipment with the following components: AS-2057 Plus JASCO autosampler, PU-2089 Plus JASCO quaternary pump, X-LC 3120 FP JASCO fluorescence detector, LC-Net II/ADC FP JASCO, and CAPCELL PAK C-18 4.6mm L-D x 25mm SHISHEIDO HPLC column. The quantification of neurotransmitters by HPLC developed a gradient method of a mixture of aqueous solutions, the mobile phase A (FMA): 2% methanol, 1% trichloroacetic acid in milli-Q water, and another organic phase, the mobile phase B (FMB): 0.1% trichloroacetic acid in acetonitrile.

First, the standard was prepared with 200 μM DOPAC, 5μM DA, 20μM 5-HIAA, 2μM 5-HT, and 25μM Trp. The calibration curve for the neurotransmitters and their metabolites was obtained. Then, 200μl of preservation buffer was added to each tissue sample. While kept on ice, it was disaggregated by sonication, in cycles of 30 seconds 2–3 times, until it was homogenized. 50 μl of perchloric acid 2.4M was added and mixed with a vortex. It was incubated for 20 minutes in the freezer. Subsequently, it was centrifuged at 1200 rpm for 20 minutes. The column was placed in the filter box, where 250 μl of FMB was added and filtered to activate the column. Then, 250μl of FMA was added to equilibrate the column. The sample was filtered without collecting it. Then, 250 μl of 30% FMB was filtered and collected. Finally, 100 μl to 150 μl were taken and placed in the injection tubes. The programming of the injection and reading of the samples, as well as the analysis of the chromatograms, was carried out with the software program ChromNAV Version 2 of the JASCO brand at the Instituto Nacional de Psiquiatría Ramón de la Fuente Muñiz (CDMX).

### 2.6 Statistics

Statistical analysis was performed in Prism Graphpad 8.3 software. The ANOVA statistical test was applied for the comparison of independent groups, followed by the *post-hoc* Tukey test to evaluate differences between sexes and differences between the control vs BBP groups. We considered statistically significant differences when P< 0.05.

## 3 Results

### 3.1 BBP-exposed offspring exhibit reduced weight

**Figure 2:**
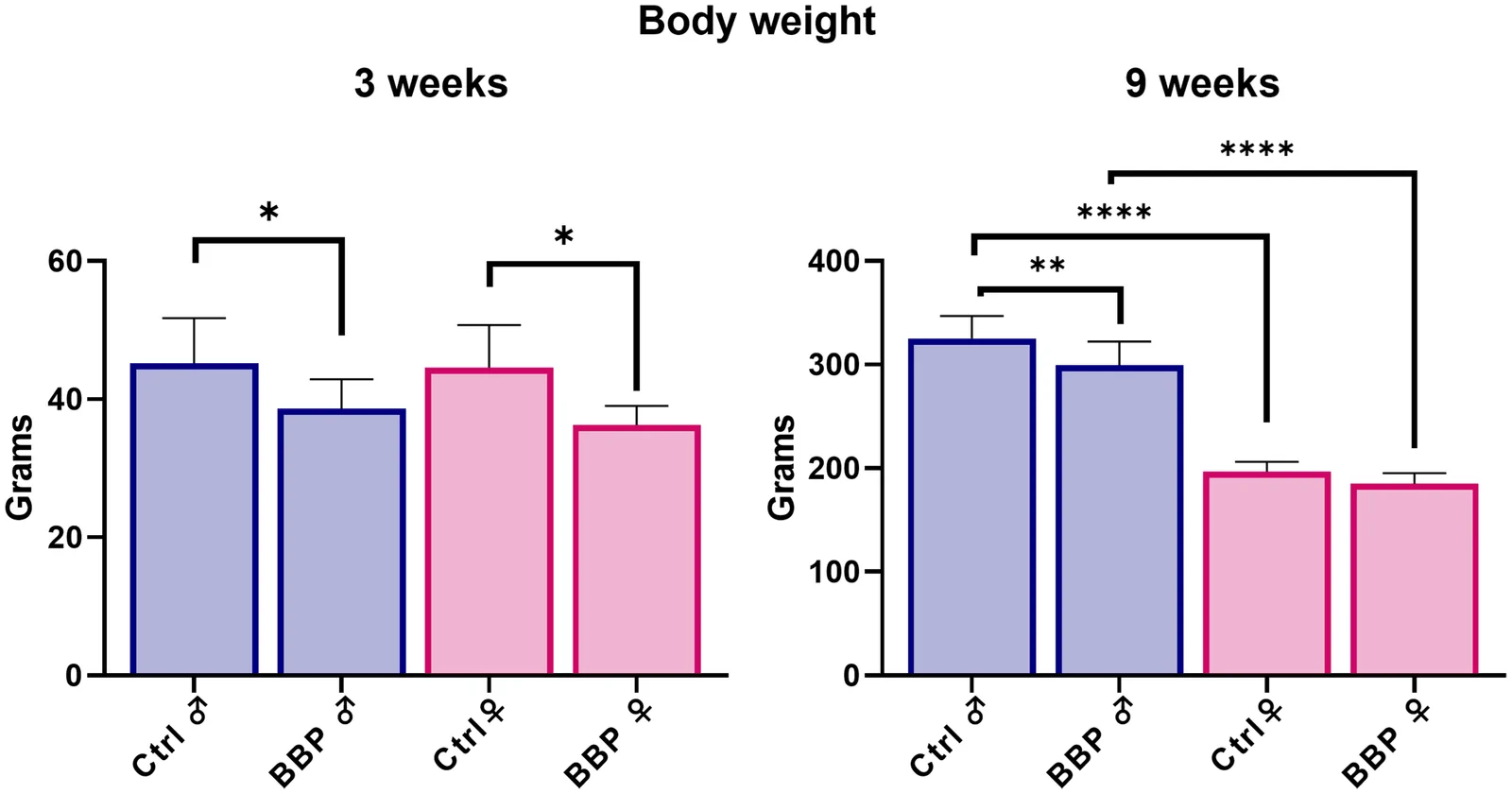
Body weight at 3 weeks (Day of weaning) and 9 weeks of age, comparison by sex. Mean ± SD is shown. *P<0.05, **P<0.01, ***P<0.001., ****P<0.0001.

Rats exposed to BBP during the perinatal period exhibited a reduced body weight at three weeks of age, coinciding with weaning (p<0.05 for males; p<0.01for females). In males, this effect persisted into adulthood (p<0.05), with an average weight of 325.3 ± 21.5 g in control rats compared to 299.2 ± 22.8 g in the BBP group (**Figure 2**).

**Figure 3:**
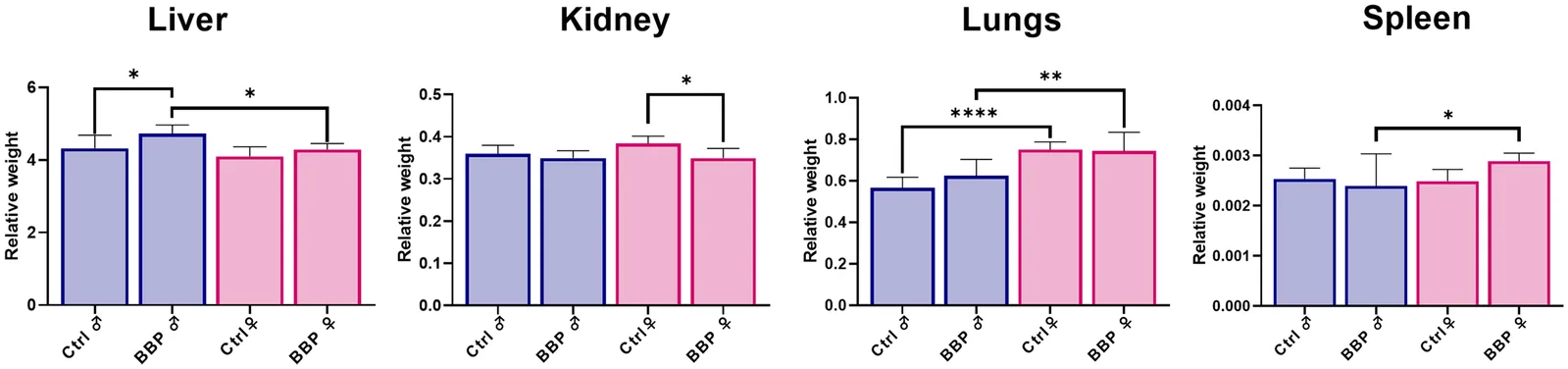
Relative organ weights of liver, kidney, lung, and spleen at sacrifice. Mean ± SD is shown. *P<0.05, **P<0.01, ****P<0.0001.

At sacrifice, the liver, kidney, lung, and spleen were removed and weighed (**Figure 3**). The net weight of each organ was then corrected relative to the total weight of each rat. The relative weight of the liver was higher in BBP-exposed (p<0.05), in contrast to the relative weight of the kidneys, which was lower in BBP females (p<0.05). About the relative weight of the lungs, it was observed that the lungs of female subjects were heavier than those of male subjects, irrespective of whether they had been exposed to BBP during the perinatal stage (p<0.01). As for the spleen, there is no change in the weight of the exposed groups, although the spleen weight of BBP females was greater than that of BBP males, a dimorphic difference that was not observed in the controls.

### 3.2 Sexual dimorphism in hormone levels in control and BBP-exposed animals

**Figure 4:**
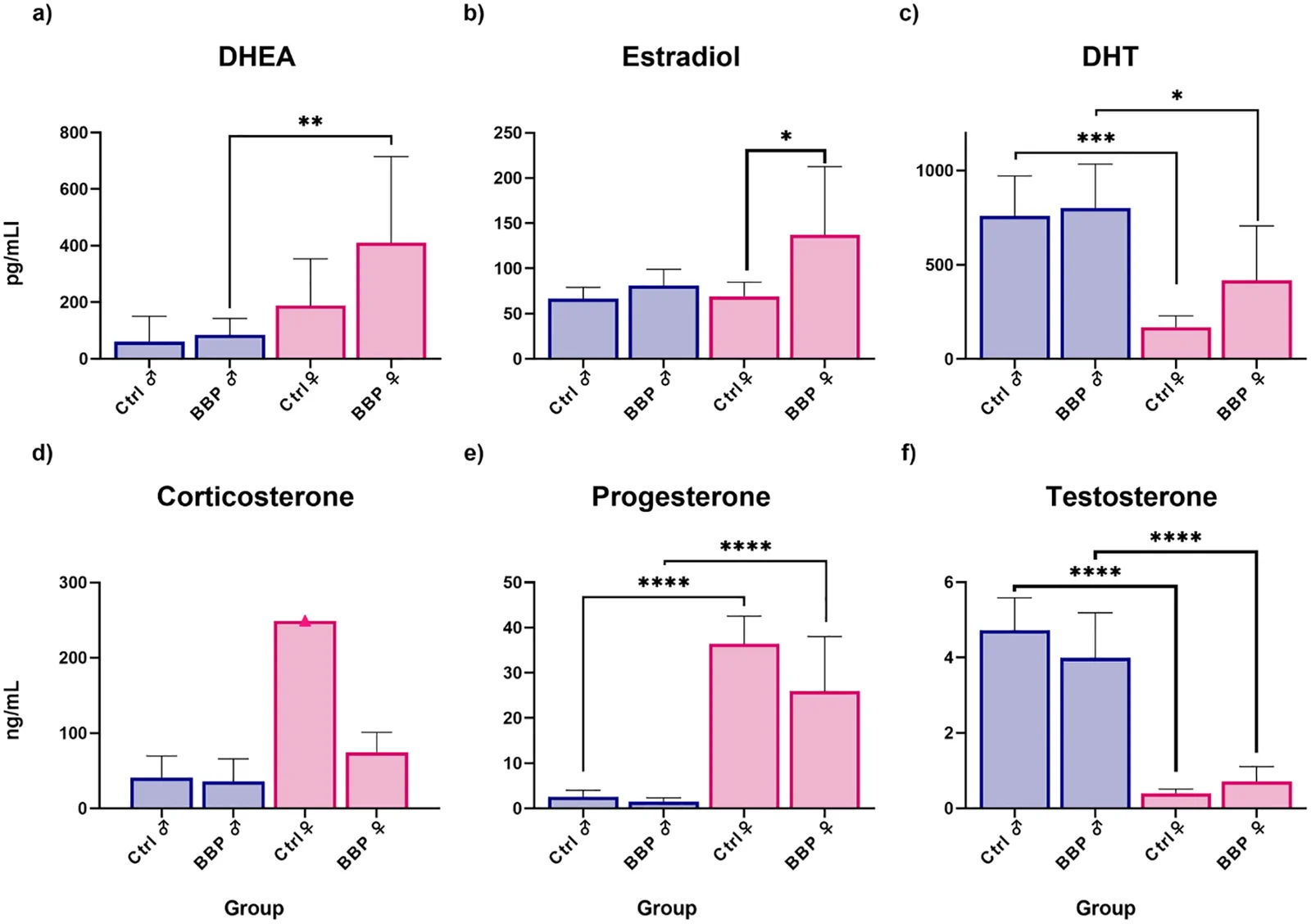
Serum steroid hormones. Comparison by sex. DHEA: dehydroepiandrostenedione, DHT: dihydrotestosterone. ▲ Quantification were higher than the sensitivity range in corticosterone females. Mean ± SD is shown. *P<0.05, **P<0.01, ***P<0.001., ****P<0.0001.

The steroid hormones DHEA, Testosterone, DHT, 17β−estradiol, Progesterone, and Corticosterone were measured in the serum of the rats (**Figure 4**). Testosterone, DHT, and Progesterone hormone levels show sexual dimorphism in both control and BBP-exposed groups, with testosterone and DHT being higher in males (p<0.0001 in both cases for T; p ≤ 0.01 for DHT) and progesterone higher in females (p<0.0001 in both cases). DHEA showed dimorphism only in the BBP-exposed group, being higher in females (p<0.01). BBP group also presented higher estradiol concentration (p<0.05). Males exposed to BBP showed no alteration in hormone levels. Corticosterone particularly showed values higher than the detection limit in most individuals, including all control females, with values higher than 240 ng/ml. Due to the absence of exact values, it was not possible to conduct statistical analyses.

### 3.3 BBP-exposed animals showed dimorphic differences in innate and adaptive immune populations

**Figure 5:**
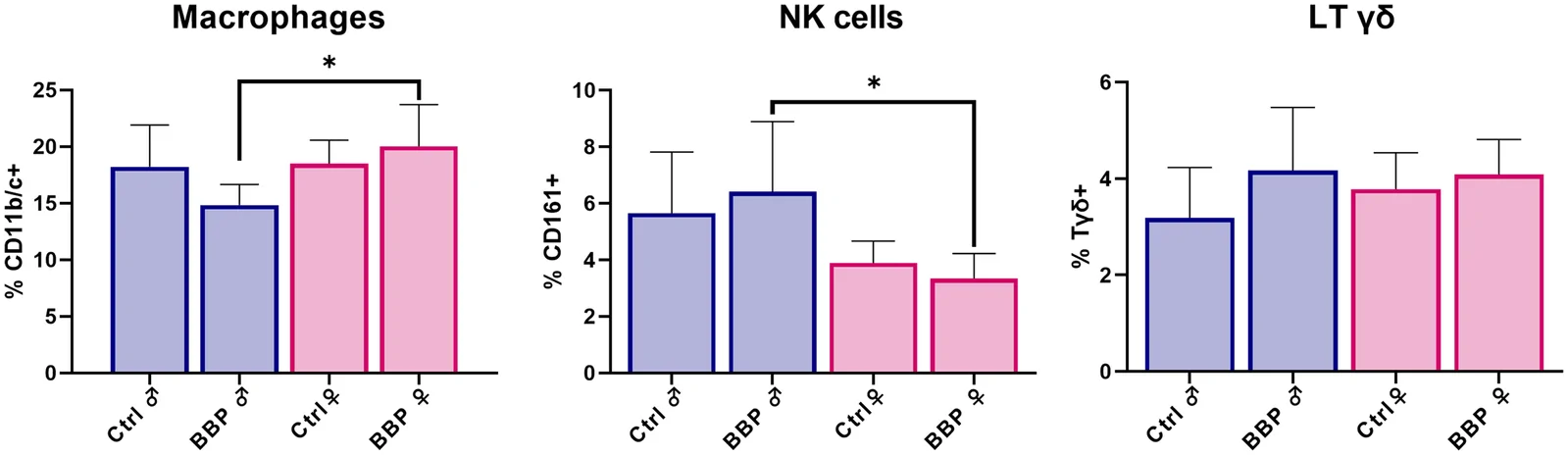
Percentage of innate immune cells. Macrophages, NK cells (CD161+) and Tγδ (γδ+) lymphocytes in the spleen. Mean ± SD is shown. *P<0.05.

In the spleen, the percentage of various immune populations was quantified. NK cells (CD161+) and macrophages (CD11b/c+) do not change in BBP-exposed rats. However, while control rats show no sexual dimorphism, BBP females exhibit a lower percentage in NK cells (p<0.05) and a higher percentage in the number of macrophages (p<0.05). Tγδ lymphocytes did not show percentage modifications (**Figure 5**).

**Figure 6:**
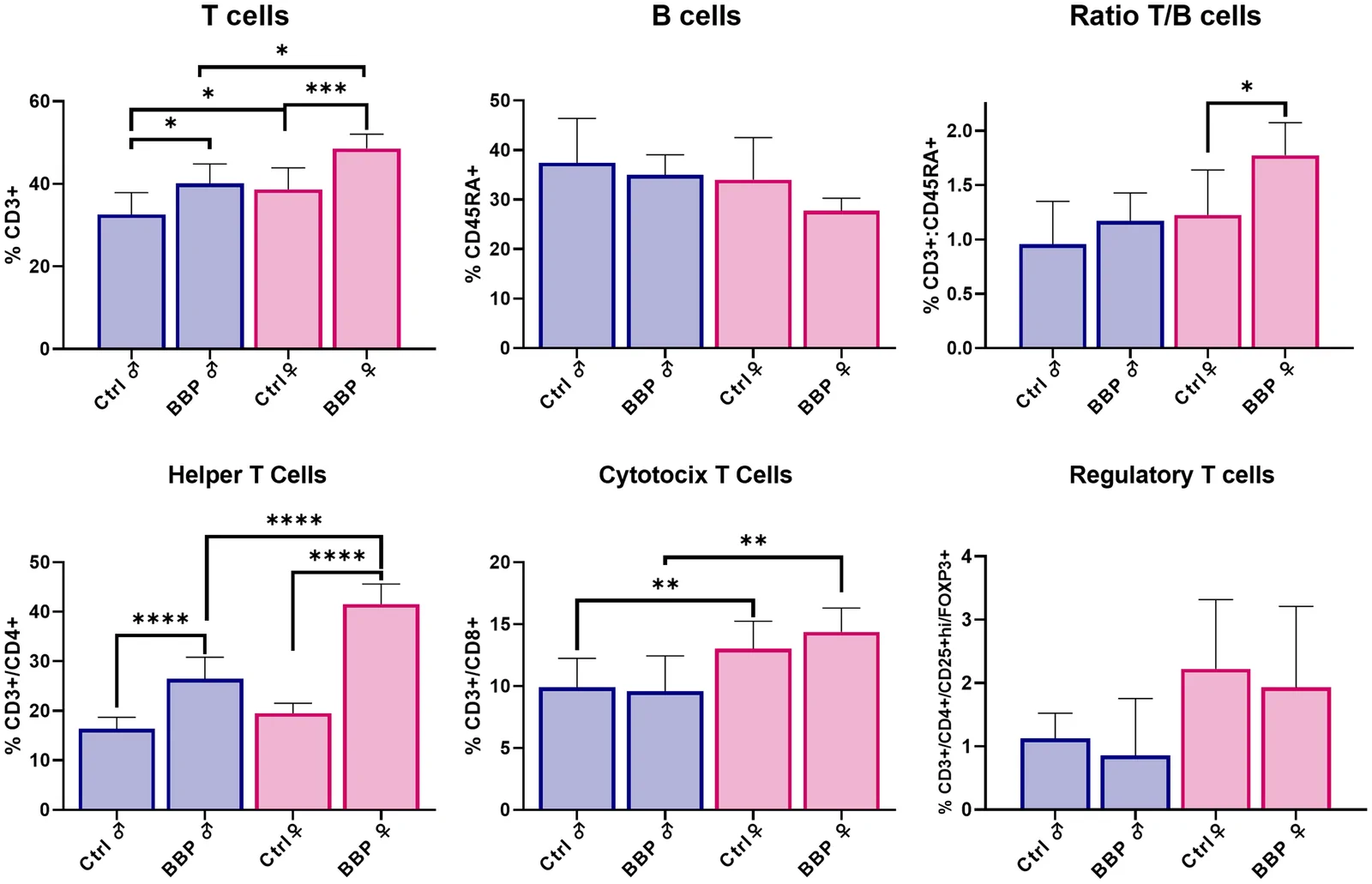
Percentage of adaptive immune cells. T cells (CD3+) and B cells (CD45RA+) immune populations in the spleen. The ratio CD3+/CD45+ was calculated. Subpopulations of T cells in the spleen were also quantified. Mean ± SD is shown. *P<0.05, **P<0.01, ***P<0.001, ****P<0.0001.

The total number of T lymphocytes CD3+ (LTs) increased in the BBP-exposed groups (p<0.05 for males; p<0.001for females), preserving the dimorphic difference regardless of treatment. B cells (CD45RA+) showed no effect due to treatment, nor for sexual dimorphism. When calculating the CD3+/CD45RA+ ratio, BBP females show a statistically significant difference with an increase in T cells over B cells (p<0.05) (**Figure 6**). The subpopulation of helper T lymphocytes CD4+ (LTh) is higher in male and female rats exposed to BBP (p<0.0001). The subpopulation of cytotoxic T lymphocytes CD8+ (LTc) shows sexual dimorphism with a higher percentage in females (p<0.005), without changes associated with perinatal BBP. From the LTh subpopulation, the percentage of CD4+/CD25hi+/FOXP3+ regulatory type cells were quantified, with no changes associated with sex or treatment.

### 3.4 Dimorphic differences were determined in cytokine levels in BBP-exposed animals

**Figure 7:**
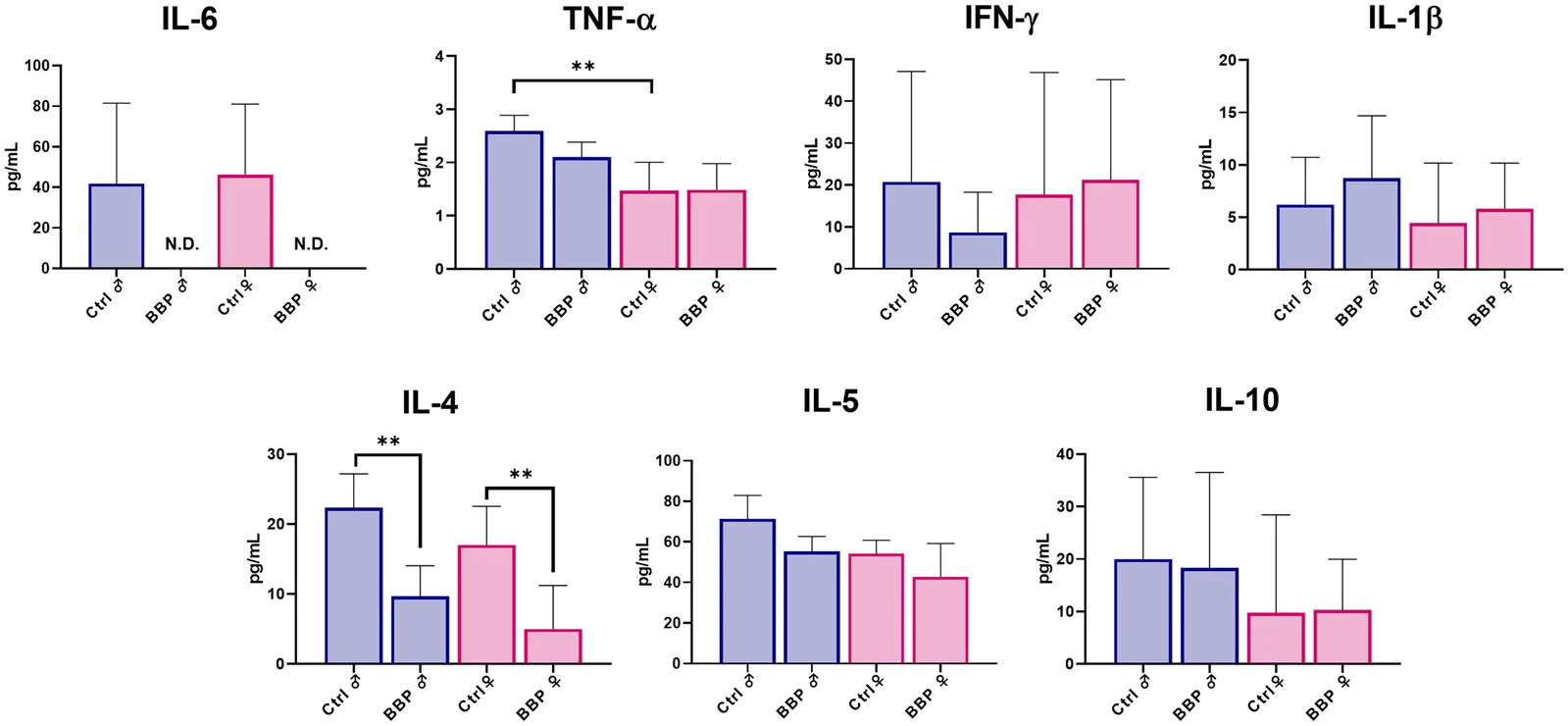
Serum cytokine concentration. N.D.: Not detectable. Individual values are shown, as well as mean ± SD. **P<0.01.

**Figure 8:**
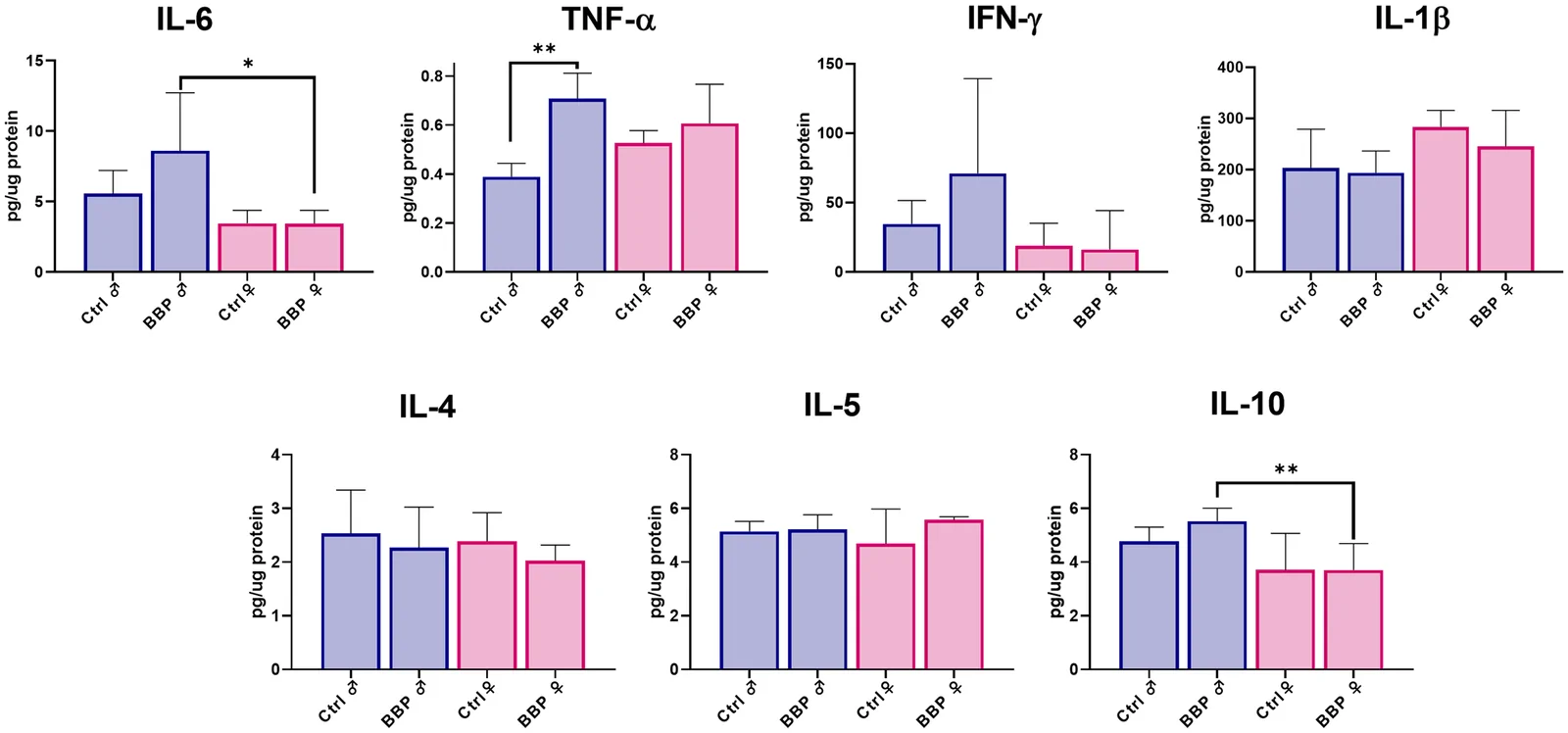
Splenic cytokine concentration. Mean ± SD is shown. *P<0.05, **P<0.01.

**Figure 9:**
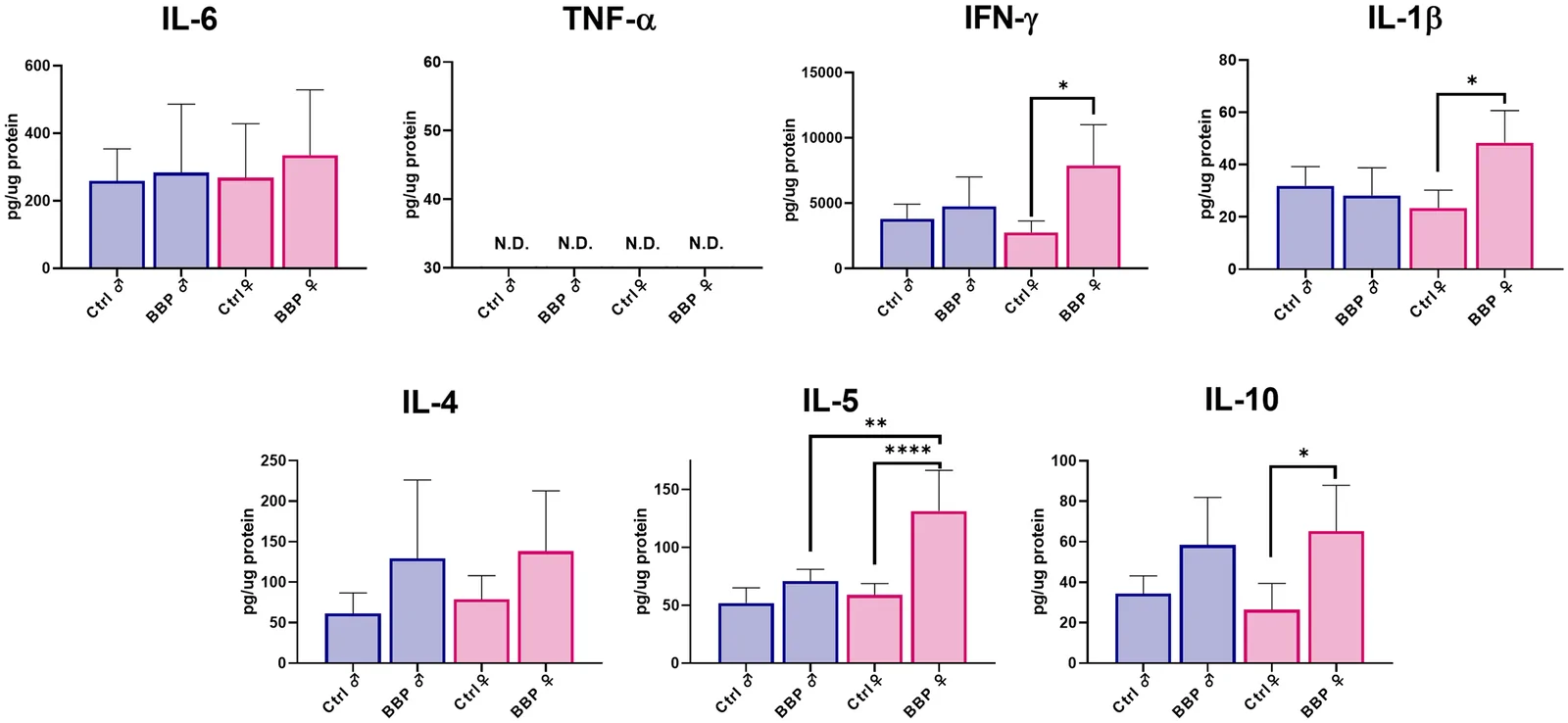
Hippocampal cytokine concentration. N.D.: Not detectable. Mean ± SD is shown. *P<0.05, **P<0.01, ****P<0.0001.

Cytokine concentration was quantified in serum (**Figure 7**), spleen (**Figure 8**), and hippocampus (**Figure 9**). Rats perinatally exposed to BBP presented undetectable levels of IL-6 and lower levels of IL-4 than Ctrl rats in serum. TNF-α showed a dimorphism percentage in the control groups, being higher in males than females (p<0.01); such differences were not significant in the BBP group. In the spleen, an increase in TNF-α concentration was observed in BBP males (p=0.002).

In the hippocampus of BBP females, an elevation in proinflammatory cytokines (e.g., IFN-γ, IL-1β, and IL-5) and a regulatory cytokine (e.g., IL-10) (p<0.01, p<0.01, p<0.0001, p<0.05, respectively). This finding indicates a state of neuroinflammation. In this case, TNF-α was undetectable (**Figure 9**).

### 3.5 Diminished levels of NTs were detected in male BBP-exposed animals

**Figure 10:**
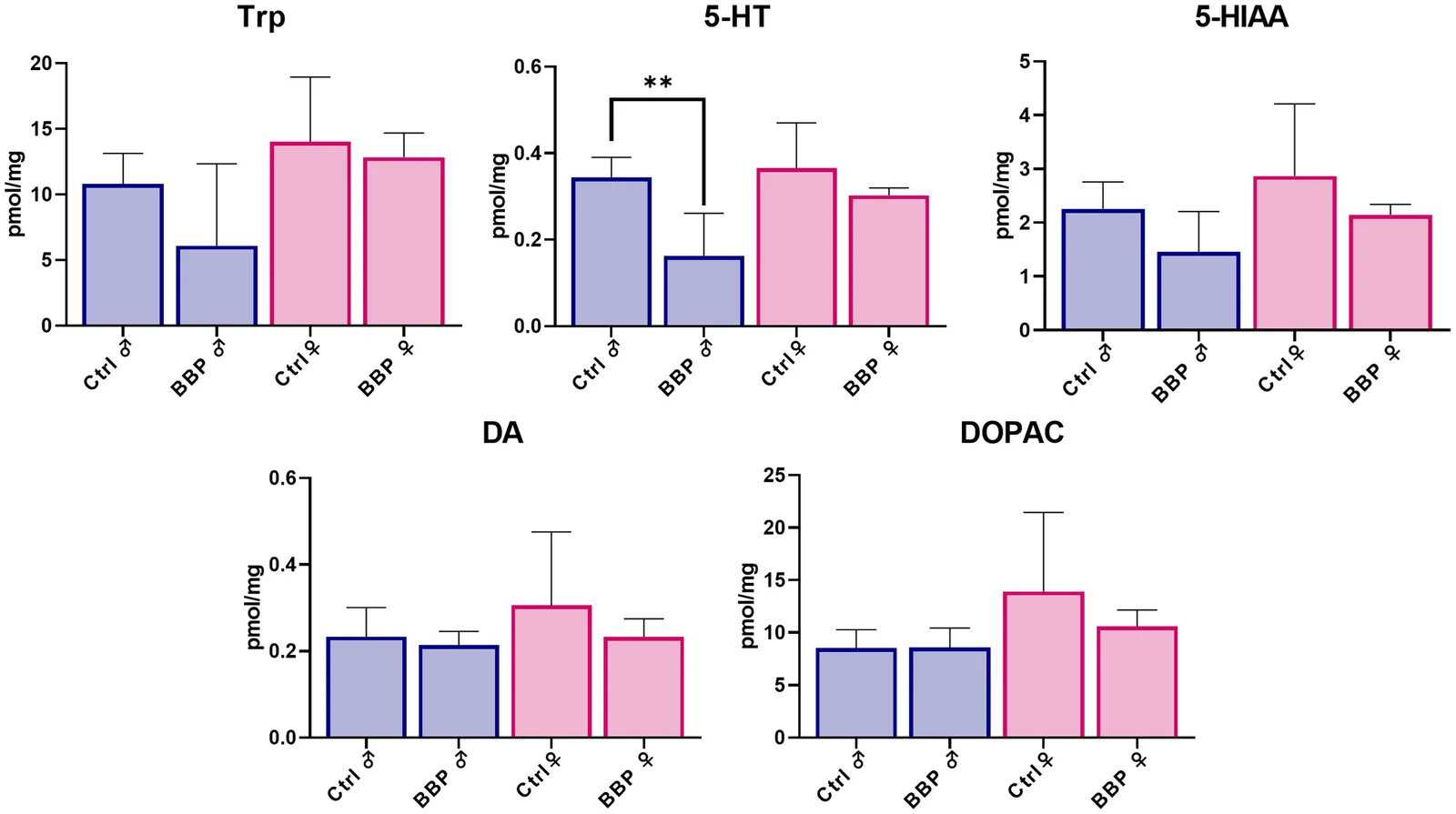
Concentration of hippocampal neurotransmitters. The amino acid Trp, Serotonin (5-HT), 5-HIAA, Dopamine and DOPAC were measured. 5-HT: 5-Hydroxy tryptophan, 5-HIAA: 5-hydroxyindole acetic acid, DOPAC: 3,4-dihydroxyphenylacetic acid. Mean ± SD is shown. **P<0.01.

The amino acid tryptophan (Trp), the neurotransmitters serotonin (5-HT) and dopamine, as well as their respective metabolites, namely 3-hydroxytyramine (DOPAC) and 5-hydroxyindoleacetic acid (5-HIAA), were also quantified in the hippocampus. (**Figure 10**). BBP males presented lower serotonin concentrations than control males (p<0.01). For the rest of the molecules, no sexual dimorphism differences or differences associated with perinatal exposure were found.

## 4 Discussion

In the present study, we evidenced that exposure to BBP dissolved in water with a concentration as low as 50 μg of BBP per liter (~13 μg/kg/day via oral) during critical development stages is sufficient to induce persistent and sex-specific alterations in the NIE network. This is consistent with research on other phthalates, where gestational and lactational exposures have been shown to impact offspring development, including neurodevelopmental trajectories (50). These findings are particularly concerning given that early life exposure to phthalates is common, and there are no international regulations regarding their use or exposure, nor procedures for their elimination from the environment.

BBP-exposed pups had lower body weights at 3 weeks of age, and this was still the case for males at 9 weeks. In humans, a correlation has been reported between BBP metabolites and lower weight, as well as a lower percentage of fat and lean mass at birth and 5 months of age, especially in males (51). Accordingly, in a longitudinal study (52), neonates exposed to BBP and other phthalates showed low birth weight, but at 3–4 years, their adiposities were higher, suggesting that phthalates would be altering metabolism and growth processes. Additionally, to the estrogenic or anti-androgenic activity, disruption of thyroid and growth hormones could be contributing to the observed alterations (53).

To evaluate possible alterations in other organs, the lung, spleen, liver, and kidney were extracted, and their weight was compared with that of control groups. In males, the liver showed a higher relative weight. In females, the kidney had a lower relative weight. Both liver and kidney contain enzymes that metabolize BBP and are organs where its metabolites accumulate. (54), whose presence would explain the weight gain or the possibility that they damage the tissue and, on the contrary, lead to a decrease in weight. Other studies have found alterations in the weight of both organs, with differences depending on the dose and the model used (55).

Given that BBP is classified as an endocrine disruptor, we evaluated the concentrations of circulating steroid hormones in serum. Testosterone, DHT, and progesterone showed dimorphic concentrations, with no alterations linked to BBP. Only estradiol concentrations in females were increased in the experimental group. The low dose used in this study was not sufficient to cause the decrease in androgens that have been previously reported with doses higher than 500 mg/kg/day (56). Finally, the amount of corticosterone, a hormone released in stressful situations in rats, was measured. However, several rats presented levels above the upper limit of detection (240 ng/ml), so we cannot associate BBP with changes in this hormone. These results do not rule out that BBP at low doses has effects on the endocrine system since the expression of hormone receptors or hormone-dependent functions was not evaluated. For instance, in a similar study, Sprague Dawley rats were exposed to BBP 10μg/mL and, at PND 65, the ERα protein levels of the amygdala were lower than controls but only in females. Thus, ER regulation could be specific for different brain structures, and higher concentrations would be needed to detect changes in the brain endocrine system.

It was observed that the immune system is susceptible to BBP. Cell populations of the spleen, a secondary lymphoid organ that mediates immune responses at the systemic level, were quantified. In the BBP groups, the number of LTs increased, especially in the female group. The percentage of CD4+ LTh was also higher in both sexes. Thus, BBP would be modulating the LT differentiation towards a helper profile rather than cytotoxic. Accordingly, it has been reported that BBP exposure promotes through epigenetic changes the exacerbation of CD4+ LTh-associated inflammation and the differentiation to the Th2+ inflammatory phenotype (42).

As for the function of the immune cells quantified, we measured some cytokines that they release, both in serum and spleen. At the peripheral level, no elevated proinflammatory cytokines were found, although IL-6 was undetectable, and IL-4 was lower in BBP rats. Because our experiment involved healthy subjects, it was expected that their cytokine levels would be lower or similar to those of the control group. However, IL-6 is a potent regulator of the immune response, so low levels of IL-6 could suggest an immunodeficiency. (57). Indeed, IL-6 plays a key role in other processes and systems (58), so IL-6 deficiencies could also lead to systemic alterations. We measure cytokines from the spleen to identify activity related to a systemic promotion of inflammation from this secondary immune organ. Particularly in the spleen of exposed males, TNF-α had a higher concentration. As evidenced by intergenerational experiments, BBP is capable of increasing inflammation of type Th2/Th17 in subjects with asthma (42, 59), and TNF-α is a key promoter of the Th2 immune response (60). In such a case, prenatal and perinatal exposure resulted in higher levels of IL-5 and IL-13 in the spleen and lymph nodes. Additionally, BBP promotes oxidative stress, IgE release, and the activation of the NF-κB signaling pathway, which also could lead to the TNF-α and other cytokines, and aggravating allergic asthma airway symptoms, including airway remodeling, hyperresponsiveness, and inflammation (59). Regarding our results, at the systemic level, we did not find inflammation markers except for TNF-α, but it is important to emphasize that we used a low concentration, and the mentioned alteration could require higher exposure to the phthalate.

In the present study, we did not administer an immune challenge that could show the consequences of the changes found. Moreover, few studies have been conducted to evaluate the effect of perinatal BBP on adult immunity. However, research has demonstrated that prenatal exposure to the phthalate DEHP can induce epigenetic changes in genes associated with the immune system, utilizing a rat model (61). In mice, the induction of Rheumatoid Arthritis worsened in those subjects that were exposed to BBP perinatally, showing higher prevalence, exacerbated symptomatology, and more secretion of inflammatory cytokines by splenocytes (62). In accordance, observational studies have evidenced associations between phthalate exposure during the second trimester of pregnancy with a higher risk of atopic dermatitis symptoms in males and a lower risk in women, according to evaluations during childhood (63). Similarly, based on the cohort of Alberta Pregnancy Outcomes and Nutrition (APrON), some correlations were found between the concentration of urine prenatal metabolites of certain phthalates and allergic conditions. For example, MBzP was associated with higher odds of developing eczema and asthma (64). Altogether, the data suggest that phthalates alter the immune system, promoting type 1 hypersensitivity.

It has been suggested that plasticizers can cross the blood-brain barrier (65) and, specifically, the hippocampus is affected by BBP and other phthalates (47, 66). Following the immune alterations found previously, cytokines were also quantified in the hippocampus. Proinflammatory cytokines IL-1β, IFN- γ and IL-5 were found to be elevated in females, as well as IL-10, a regulatory cytokine that could be compensating for inflammation. In contrast, males did not present any cytokine elevation, suggesting that the female hippocampus would be more susceptible to neuroinflammation. An important mechanism by which phthalates cause damage is oxidative stress accompanied by inflammation (67). In other models, proinflammatory cytokines have been reported (68, 69) together with neurochemical changes, although dimorphic differences have not been evaluated.

Males presented decreased serotonin levels in the hippocampus. Low serotonin levels in adult male mice exposed to BBP (50, 250, and 1250 mg/kg/day for 14 days) at 4 weeks old had already been reported (47). In that study, elevated ROS levels, lower CREB phosphorylation, depression-like behaviors, and worse performance in memory tasks were also found. Our results suggest that the effect of BBP at the central nervous system level can be observed even when exposure was in perinatal life and at lower doses. In adulthood, the decrease of serotonin in the hippocampus would have repercussions on learning and memory, in addition to predisposing to affective disorders. Conversely, in this case, serotonin levels were not altered in females, reinforcing the dimorphic susceptibility to the BBP exposure during perinatal stages.

Perinatal exposure to phthalates leads to molecular, structural, and behavioral alterations, including a reduction in the number of neurons and synapses as well as an increase in the size of the medial prefrontal cortex in correlation with a deficit in cognitive flexibility (70). After exposure to a mixture of phthalates, pesticides, and BPA, males showed dose-dependent increases in locomotion and reduced social interaction. Both sexes increased struggling in the forced swim, suggesting hyperactivity and social deficits are more present in males. In the same study (71), transcriptional changes were measured in different brain structures such as the hippocampus, hypothalamus, amygdala, and prefrontal cortex, where most modifications were significant only in males. The latter study demonstrates that even when some behavioral alterations could be similar in both sexes, the biochemical pathways involved could differ. In humans, exposure to phthalates is associated with smaller total gray matter (monoethyl phthalate) and cerebral white matter in girls (monoisobutyl phthalate) with lower child IQ (72).

There are some reports of dimorphic effects in the nervous system and behavior regarding perinatal exposure to BBP. For instance, exposure to BBP (25mg and 750 mg/kg/day) resulted in dimorphic metabolomic changes in the brain with amino acid alteration for both sexes and phospholipid metabolism perturbations only in males. Interestingly, only males showed metabolomic changes with the low BBP dose, and such changes were more than those in the high dose group (45). In another study, female rats showed changes in proteins of the amygdala required for normal learning and memory behaviors, and accordingly, they showed learning alterations in the fear conditioning task, meanwhile male didn’t (73). Besides, there is epidemiological evidence for an inverse correlation between higher BBP exposure and worse psychomotor development only in girls (50). Meanwhile, phthalate exposure would be related to worsened cognitive functions, but females would be more resilient to the toxicity effects, especially at a younger age and/or low doses of exposure (74). The dimorphic differences could be associated with neurodevelopmental differences and masculinization/defeminization processes during early brain development; however, there are no studies that confirm such hypotheses.

## 5 Conclusions

Exposure to BBP during gestation and lactation causes long-term dimorphic changes in the NIE network, even when exposure levels are within the range considered safe. Low doses of BBP were not enough to replicate hormonal alterations found in studies with high doses of exposure, but splenic immune cell composition was modified in both sexes with a higher LT helper population. Regarding the nervous system, the hippocampus had dimorphic consequences. Meanwhile female hippocampus showed neuroinflammation, and males had lower serotonin levels. It is necessary to consider the effects of BBP plasticizer at critical periods of development, especially at the molecular level, to determine its toxicity and thereby promote modifications to its use and exposure regulation.

## Data Availability

The data generated in this study are available upon request from the corresponding author.
